# A preliminary investigation into the prevalence and prediction of problematic cell phone use

**DOI:** 10.1556/JBA.3.2014.004

**Published:** 2014-02-03

**Authors:** Peter Smetaniuk

**Affiliations:** San Francisco State University, San Francisco, CA, USA

**Keywords:** technological addictions (TA), problematic mobile phone usage, behavioral addictions

## Abstract

*Background and aims:* Likening mobile phone use dependency to the classification of excessive behaviors may be necessarily equivalent in seriousness to previously established addictions such as problematic computing or excessive gambling. The aim of the study explores into the behavior of excessive use of mobile phones as a pathological behavior. *Methods:* Two studies investigated criteria for problematic mobile phone usage by examining student (Study 1, *N* = 301) and nonstudent (Study 2, *N* = 362) responses to a set of adapted mobile phone addiction inventories. Study 1 investigated cell phone addiction inventories as constructs designed to measure problematic cell phone use. Additionally, Study 2 sought to predict age, depression, extraversion, emotional stability, impulse control, and self-esteem as independent variables that augment respondents’ perceptions of problematic use. *Results:* The results from Study 1 and Study 2 indicate that 10 to 25% of the participants tested exhibited problematic cell phone usage. Additionally, age, depression, extraversion, and low impulse control are the most suitable predictors for problematic use. *Conclusions:* The results of the two studies indicate that problematic mobile phone use does occur and ought to be taken seriously by the psychological community. Presently, there is limited data providing conclusive evidence for a comprehensible categorization of cell phone addiction, as well as a unified explanatory model specific to problematic mobile phone use. Studies such as this one may contribute substantial findings, adding scientific significance, and offering a valuable submission for the ongoing progress of creating intervention frameworks relative to “virtual addictions”.

## Introduction

With the expanse and advent of virtual technologies, mobile cellular devices are no longer considered basic phones. To date, additional features in most cell phones now let phone owners’ access to the Internet (i.e., smartphones, BlackBerries, and iPhones). With the relatively “new” condition of Internet access mobility, can people become addicted to their cell phones (Suratt, 2006), and if so, what are the prevailing consequences? More so, what diagnostic criteria ought to be established in explaining problematic cell phone use and what psychometric inventories can be implemented to determine, diagnose, and predict problematic mobile phone use?

Griffiths (2000) best sums up excessive and appetitive behavior relative to information and communication technologies (i.e., a social pathology specific to cyberspace): “Technological addictions (TA) are operationally defined as nonchemical (behavioral) addictions, which involve human-machine interaction. They can either be passive (e.g., TV) or active (e.g., Computer games), and usually contain inducing and reinforcing features which may contribute to the promotion of addictive tendencies” (p. 211). Furthermore, TAs can best be described as a subset of behavioral addictions (Griffiths, 2000) and relative to problematic mobile phone use this model is comprised of discrete components such as salience, or preoccupation (i.e., when an individual is not engaged in cell phone use, he or she will have thoughts or cravings about the next opportunity to use their phone); mood modification (i.e., an emotional response as a consequence of cell phone use, which can be seen as a coping strategy for the individual who experiences a “high” or euphoric feelings of “escape”); tolerance (i.e., increasing amounts of cell phone activity are necessary to achieve the former effect level); withdrawal (i.e., unpleasant physiological or subjective experiences such as moodiness, irritability, and anxiousness when cell phone use is discontinued or immediately reduced); conflict (i.e., interpersonal and/or intrapsychic dissension, such as losing one’s job or relationship due to cell phone use); and relapse (i.e., when reverting back to “old habits” or a return to addictive behaviors after a temporary improvement).

Presenting the consequences of behavioral addictions by exploring measures, determinants, and social consequences associated with the pathological use of information and communications technologies (ICT), this study focuses on the aforementioned criteria for impulse control disorder (ICD), and based on the Diagnostic and Statistical Manual of Mental Disorders (APA; DSM-IV, 2000) as it relates to problematic cell phone use. Additionally, this study proposes that problematic mobile phone use may have similar patterns in behavior to impulse control disorders (ICD) such as pathological gambling (Grant, Potenza, Weinstein & Gorelick, 2010). Similar to other taxing behaviors such as compulsive buying and sexual addiction, “problematic mobile phone use” has yet to be considered as a separate behavioral disorder for inclusion in the DSM.

Many ICT investigations – which are currently ongoing – focus on the “overuse” of virtual technologies (VT), and because of the complex nature of behavioral addiction (DiClemente, 2003), the frequency of and inappropriate use of cell phone use are only two out of many determinants for assessing and concluding that the VT behavior is an addiction. Other independent variables such as age, personality traits (e.g., extraversion, neuroticism), depression, low impulse-control, and low self-esteem, for example, may contribute and mediate the problematic use of VTs.

Past studies have tested the prediction that certain personality traits, such as extraversion and neuroticism (Bianchi & Phillips, 2005), are linked to addictive behaviors. As sensation seekers, extraverts tend to express social affects more favorably, and this belief assumes that computer and cell phone use may support specific socializing interests (Griffiths, 1995). Thus, the present research expects to find the trait of extraversion to be an important predictor of higher problematic cell phone use.

The neurotic personality type is characterized by self-conscious and impulsive trait behaviors (Anastasi & Urbina, 1997). Groups scoring high on scales for neuroticism or scoring low in emotional stability are both inclined to use virtual technologies as a form of escape (Amiel & Sargent, 2004). The neurotic individual also displays overly emotional, distressed behavior and interacts with their mobile phone (and Internet) use in a unique way (i.e., mostly in a negative way), for example, as a substitute for conventional face-to-face interactions. Additionally, neurotics prefer to do less emailing and less texting, and they tend to avoid blogging or online discussion groups (Amiel & Sargent, 2004). However, in Bianchi and Phillips’ study (2005), “neuroticism” as a factor for explaining problematic mobile phone use was not evidenced by higher scores on the Mobile Phone Problem Use Scale (MPPUS). Nevertheless, high neuroticism is characterized by moodiness (i.e., experiencing a negative affect), and linked to other immoderate behaviors such as eating disorders and drug dependency (Davis & Claridge, 1998). Thus, exploration of the neurotic dimension may well be a significant predictor of problematic cell phone usage.

Undeviatingly, as a factor, low self-esteem has been associated with behavioral addiction (Marlatt, Baer, Donovan & Kivlahan, 1998). According to Baumeister (1997; Baumeister, Campbell, Krueger & Vohs, 2003), individuals expressing low self-esteem show self-defeating behaviors; but many investigations into self-esteem, correlated with other addictive behaviors, tend to show inconsistencies in their results (Baumeister et al., 2003). Therefore, interpretations of the effects are cautioned, and this caveat should be taken into consideration due to the complex nature of self-esteem. In accordance, Forest and Wood (2012) found that even though social networking sites are quite appealing to individuals with low self-esteem, their Facebook self-disclosures projected more negativity than positivity, which elicits unfavorable responses from others. Again, Bianchi and Phillips (2005) suggested that expressed low self-esteem reflects the need to seek psychological reassurance from others and these individuals are certain to use their mobile phones inaptly. Additional findings reported by Bianchi and Phillips (2005) showed that problem mobile phone use was a function of age, extraversion, and low self-esteem. Their results showed that adolescents, extraverts, and those participants with low self-esteem had scored highest on the MPPUS. The present study utilizes the MPPUS by exploring its psychometric properties relative to self-esteem and other independent variables such as age, impulse control, extra-version, neuroticism, and depression.

Definably, the DSM-IV (2000), Axis I, classifies depression as a mood disorder accompanied by subjective discomfort that hinders the sufferer’s ability to function. More specifically, and for the purpose of this study’s operational definition, depressive symptoms are those without hypomanic episodes (viz., unipolar) and are characterized by feelings of sadness, worthlessness, emptiness, and feelings of futility. Along with the feelings of depression, there are associated symptoms such as social withdrawal, low energy levels and lack of motivation (Sue, Sue & Sue, 2010). Focusing on the relationship between cell phone usage, depression, and mobile accessibility to the Internet, previous studies have shown that depressed individuals tend to frequent the Internet much more than those without symptoms (Lam & Peng, 2010; Morrison & Gore, 2010; Young & Rogers, 1998). Excessive interactive activities such as frequenting chat rooms (Bonetti, Campbell & Gilmore, 2010), gambling (Griffiths & Wood, 2000), late night Web browsing (Gangwisch et al., 2010) social networking (Michael & Michael, 2011), periodic visits to mental health-related websites (Bessiere, Pressman, Kiesler & Kraut, 2010), and video gaming (Weaver et al., 2009) are also associated with increased depressive symptoms. Hence, the existing literature reveals that there is a moderately strong relationship between depression and excessive Internet use. Since most mobile phones currently have Internet access, this study proposes that there will be similar findings between depression and mobile phone usage.

One key aspect of behavioral addiction is a lack of self-control. According to the DSM-IV (2000), impulse control disorder (ICD) “…is the failure to resist an impulse, drive, or temptation to perform an act that is harmful to the person or to others” (p. 663). Other common core qualities shared by people with low impulse control are repetitive engagements (i.e., compulsivity) in the behavior despite adverse consequences (e.g., distress and impaired functioning). Minimal control over the problematic behavior exists with an urge or craving state (i.e., psychological salience) prior to engaging in the behavior, and a hedonic quality is experienced (i.e., euphoric) during the performance of the behavior (Grant et al., 2010). Correspondingly, in a recent study by Roberts and Pirog (2013), their results indicated that impulsiveness predicted addictive tendencies in both mobile phone technology dependency and excessive instant messaging. Thus, the discrete characteristics of low impulse control are of considerable importance as components explaining problematic cell phone use.

Differences in age have also been attributed to mobile phone usage. Older individuals have shown less motivation in espousing advanced virtual technologies than do adolescents. One explanation given by Brickfield (1985) is that older individuals have less positive attitudes toward novel technology and its products. Another speculative explanation may be that older people find many of the virtual technologies somewhat overwhelming in the manner of usage and operation (i.e., learning to navigate through the many apps may prove frustrating). More recently, a study concluded that the excessive usage of cell phone activities among young adults could have a dramatic effect on health issues (Pedersen, 2012). Findings showed that young adults aged 20–24 years old who frequent online activities via computers (or smartphones) demonstrate an increase in depressive symptoms and difficulties in sleeping (Pedersen, 2012). Thus, this study expects to find that older cell phone users have fewer cell phone usage problems. Of course, this assumption ought to be addressed with caution because older cell phone users may simply perform *less frequent* activities on their cell phones, contributing to the fallacious assumption that they express fewer problematic issues.

Over the past two decades, the extent of the expression “addiction” has broadened to include any behavior comprised of having an appetitive nature, as well as including reinforcing behaviors that augment repetitive, self-destructive outcomes and in most cases, are difficult to stop (Orford, 2001). Observing problematic cell phone users, it appears and does make sense the use of mobile phones should be included into this category of appetitive behaviors – however, if and only if the cell phone behavior fulfills the required number of addiction criteria (i.e., preoccupation, mood modification, withdrawal, escapism and dysphoric relief, tolerance and conflict/loss).

Exploring the phenomenology of technological addiction and based on any of the half dozen addiction models presented by DiClemente (2003, pp. 6–18), this present study also proposes a problematic mobile phone use theoretical model explained by the coping/learning paradigm for addiction (Wills & Shiffman, 1985). This study’s proposed model’s central message is based on patterns of behavior that reflect inadequate coping strategies related to any number of daily stressors, frustration, irritability, depression, and boredom. From this theoretical viewpoint, individuals resort to using their cell phones as a substitute coping strategy and “learn” to rely on their extreme behavior or their dependency on cell phone use for moderating dysphoric experiences. This model does not claim or convey the idea that the use of cell phones “causes” the addiction, but rather that cell phone use dependency “reinforces” the excessive behavior by moderating certain negative experiences, as well as modifying dysphoric moods as they arise for the sufferer. Thus, problematic cell phone users have a direct, accessible, and effective modus operandi that induces and minimizes any adverse emotional experiences that arise. Additionally, the cell phone may be an effective distractor, shifting the user’s awareness of the dysphoric affects toward more tolerable levels of emotion. Of course, there are dozens of reasons causing addictive behaviors relative to psychological dependency (DiClemente, 2003). Nevertheless, the characteristics for almost all addiction models coincide with the American Psychiatric Association’s (2000) definition, which includes psychological salience (i.e., an urge or craving state), symptoms of withdrawal (e.g., irritability, nervousness, restlessness), and tolerance (i.e., needing more to produce the same initial effect). Based on the aforementioned symptomatic features, those individuals coping with negative psychological experiences as they occur is a function of “immediate” cell phone use engagement.

With pathological gambling classified by the DSM-IV (2000) as an impulse-control disorder, many researchers (Grant et al., 2010) have adapted and utilized criteria associated with gambling disorders for other excessive behaviors, such as compulsive buying, sexual addiction, and computer/video game playing (Goldberg, 1995; Brenner, 1997; Morahan-Martin & Schumacher, 2000). Some psychologists require multiple criteria, and that all of them meet the classification of TA, while others require only a few criteria out of many. Accordingly, Bianchi and Phillips (2005) constructed a 27-item questionnaire called the MPPUS – with scales measuring the concerns of craving and tolerance, plus conflicts in social, financial, and occupational challenges. Similarly, Young (1998, 2004) developed Internet addiction working models (i.e., diagnostic inventories) that measure the symptoms and impairments of an individual’s Internet use based on the DSM-IV (2000) criteria for substance dependence and pathological gambling. One of Young’s scales consisted of eight items: individuals were classified as “addicted” if they answered five out of the eight adapted diagnostic criteria with personal consequences ranging from sleep deprivation to ignored responsibilities, and to marital, academic, and job related problems (Young, 2004). Expanding the eight-item list of characteristics associated with problematic Internet use, Young devised 20-items (i.e., called the Internet Addiction Test, or IAT) measuring symptoms related to impulse control disorder (ICD) and dependency (Young, 1998, 2004).

## Overview

As a preliminary investigation (Study 1), this study utilizes Young’s models (i.e., questionnaires) for Internet addiction by semantically modifying the Internet-related variables to fit cell phone use. Additionally, in Study 2, and along with utilizing the MPPUS (Bianchi & Phillips, 2005), this study seeks to predict that the scores on Young’s modified addiction tests and the MPPUS will correlate significantly with scores on the extraversion, neuroticism, self-esteem, age, depression, and impulse control scales. To investigate problematic mobile phone use, two studies (Study 1 and Study 2) were conducted. Study 1 was considered a pilot study investigating cell phone addiction inventories as constructs designed to measure problematic cell phone use. Study 2 expanded on Study 1’s overall outcomes by using the questionnaires that performed well statistically in Study 1. Additionally, Study 2 included independent variables such as age, depression, extraversion, emotional stability, impulse control, and self-esteem. One interest under investigation was establishing whether problematic cell phone use exists within the student population and can be measured by adapted mobile phone addiction scales. A secondary interest considered the predictability of problematic mobile phone use with the associated dispositional and behavioral traits (i.e., independent variables aforementioned) as predictor variables. Utilizing scales addressing criteria for problematic cell phone use, clinicians and researchers can have the psychometric capability for classifying vulnerable groups needing support – as well as promoting theoretical models addressing problematic mobile phone use.

## Study 1 Method

### Study 1 participants

A total of 301 undergrad students (52 men, 249 women, *M*_age_ = 21, age range: 18–59), comprised primarily of psychology majors attending San Francisco State University (SFSU), responded to a set of inventories via an online survey process (SFSU’s Sona System). Out of 364 respondents, 301 responses were used for analysis. Sixty-three respondents were excluded because they failed to complete all of the items on the questionnaires as required (response rate = 82.69%). Because a convenience sampling method was used, the majority of the participants were students majoring in psychology; therefore, generalizability of the findings to the general population is cautioned.

### Study 1 materials and procedure

Two inventories were used: the Adapted Mobile Phone Use Habits (AMPUH), and the Adapted Cell Phone Addiction Test (ACPAT).

*The Adapted Mobile Phone Use Habits (AMPUH)*. Consisting of ten items, the AMPUH uses a 10-item bivariate scale (yes or no response). The AMPUH items were semantically modified from addressing gambling behaviors, based on the DSM-IV (2000) criteria for pathological gambling, to fit mobile phone use (see Appendix A). Item number one, for example, was semantically changed from, “Are you preoccupied with gambling?” to “Are you preoccupied with your mobile phone?” Previous studies have utilized the DSM diagnostic criteria for other addiction disorders such as video-game use (Fisher, 1994; Griffiths & Dancaster, 1995; Griffiths & Hunt, 1998), television addiction (Horvath, 2004) and Internet addiction (Young, 2004). Problematic mobile phone use exists only if the mobile phone user endorses at least half (five) of the ten items listed. A person’s mobile phone use is considered pathological (Gentile, 2009) only after it has resulted in negative consequences in discrete areas of his or her life, such as loss of a job or loss of a friendship (i.e., conflict/loss or intrapsychic dissension). Each item on the AMPUH is a behavioral characteristic associated with a symptom relative to addictive behavior (*M* = 1.46, *SD* = 0.50). Cronbach’s alpha at SFSU was acceptable: α = .75.

**APPENDIX A:** Adapted Mobile Phone Use Habits (AMPUH)

Item (Criterion)	
1.	Are you preoccupied with your mobile phone? (Salience)
2.	Does using your mobile phone help you feel relaxed? (Mood modification)
3.	Have you made repeated efforts to cut down or stop using your mobile phone? (Relapse)
4.	Are you restless or irritable when attempting to cut down? (Withdrawal)
5.	Do you use your mobile phone to escape problems or lift your mood? (E/DR)
6.	After a large mobile phone bill, do you continue to use it? (Tolerance)
7.	Do you lie to others about how much you use your cell phone? (Cognitive distortion)
8.	Have you ever committed acts (theft) to finance your use of your cell phone? (Resort to ASB)
9.	Has your mobile phone caused you to lose a significant other or job? (Conflict/Loss)
10.	Do you rely on others to relieve financial problems caused by using your mobile phone? (Desperation)

*Note:* ASB denotes “antisocial behavior”. E/DR denotes “Escapism/Dysphoric Relief. Cognitive distortion denotes lying and concealing to others the extent of the dependency. The AMPUH is a ten-item inventory with a dichotomous scale (either a *yes* or a *no* response). All items are semantically modified to fit mobile phone use. Originally, all items were taken from the DSM-IV criteria for pathological gambling. From “Diagnostic criteria for 312.31 Pathological gambling”, APA: *Diagnostic and Statistical Manual of Mental Disorders,* 4th Ed. DSM-IV-TR (2000). Reliability: AMPUH_sp_ (student pop.) *a* = .75; AMPUH_ap_ (adult pop.), *a* = .79.

*The Adapted Cell Phone Addiction Test (ACPAT).* Consisting of 20 items (see Appendix B), the ACPAT was originally designed to measure Internet addiction (Online Addiction Test developed by Young, 1998). In this study, the items on this questionnaire were semantically modified to fit cell phone use. For example, question number 14, “How often do you lose sleep due to late-night computing?” was modified to “How often do you lose sleep due to late-night phone use?” Each item was measured on a 5-point Likert scale ranging from 1 = rarely to 3 = frequently to 5 = always. The participant’s level of addiction is computed by the total sum of all of the scores: scoring within the range of 20 to 49 indicates a low-to-moderate degree of concern; scoring within 50 to 79 indicates a moderate-to-high degree of concern; and scoring within 80 to 100 indicates a severe level of concern, whereby problematic use should be addressed. With a Cronbach’s alpha of .93 at SFSU, the ACPAT exhibits excellent internal consistency and its reliability is highly satisfactory (*M* = 33.45, *SD* = 0.69). Relative to problematic Internet use, the ACPAT addresses six factors such as preoccupation (salience), excessive use, neglecting work, anticipation, lack of control and neglecting social life (Wydanto & McMurran, 2004). With the added feature of Internet access on most mobile phones, the present study expects to find similar results in the ACPAT relative to problematic cell phone use.

**APPENDIX B:** Adapted Cell Phone Addiction Test (ACPAT)

Item	
1.	How often do you find that you stay on your cell phone longer than you intended?
2.	How often do you neglect household chores to spend more time on your mobile phone?
3.	How often do you prefer the excitement of your mobile phone use rather than to intimacy with your partner?
4.	How often do you form new relationships with mobile phone callers?
5.	How often do others in your life complain to you about the amount of time you spend on your mobile phone?
6.	How often do your grades or schoolwork suffer because of the amount of time you spend on your mobile phone?
7.	How often do you check your incoming messages before something else that you need to do?
8.	How often does your job performance or productivity suffer because of your mobile phone use?
9.	How often do you become defensive or secretive when anyone asks you what you do on your mobile phone?
10.	How often do you block out disturbing thoughts about your life with soothing thoughts of using your mobile phone?
11.	How often do you find yourself anticipating when you can use your mobile phone?
12.	How often do you fear that life without your mobile phone would be boring and joyless?
13.	How often do you snap, yell, or act annoyed if someone bothers you while using your mobile phone?
14.	How often do you lose sleep due to late-night phone use?
15.	How often do you feel preoccupied with your cell phone even when it’s off, or fantasize about being connected to someone?
16.	How often do you find yourself saying, “Just a few more minutes,” when on your mobile phone?
17.	How often do you try to cut down the amount of time you spend on your mobile phone and fail?
18.	How often do you try to hide or invent excuses as to how long you’ve been on your mobile phone?
19.	How often do you choose to spend more time on your mobile phone over going out and spending time with others?
20.	How often do you feel depressed, moody, or nervous when you are not using your mobile phone, but then the feeling goes away once you’re back to using it?

*Note:* The 20-item questions were semantically modified to fit mobile phone use. Responses are measured on a five-point scale where 1 = Rarely, 2 = Occasionally, 3 = Frequently, 4 = Often, and 5 = Always. Scales measure *low-to-moderate, moderate-to-high,* and *severe* levels of mobile phone use dependency. Originally developed by Young (2004) for measuring Internet addiction and based on DSM-IV criteria for “pathological gambling”. Reliability: ACPATsp (student pop.) *a* = .93 ; ACPAT_ap_ (adult pop.), *a* = .96.

### Ethics

The study procedures were carried out in accordance with the Declaration of Helsinki. The Institutional Review Board of San Francisco State University approved the study. All participants were provided an “informed consent” of the study.

## Study 1 Results

### AMPUH results

Table 1 shows that on the AMPUH_sp_ (sp = student population), the respondents answered the required number of items with “yes” responses (*N* = 301) with 19.92% of the respondents scoring five or more, and therefore satisfying the required number of criteria for indicating symptoms of behavioral addiction. Most notably, endorsements on the AMPUH_sp_ showed relatively moderate to high percentages of the participants’ “yes” responses reflecting criteria for salience (52%), mood modification (47%), relapse (32%), withdrawal (20%), escapism/dysphoric relief (47%), and tolerance (60%). The overall findings suggest that the AMPUH_sp_ is a reliable inventory for assessing problematic cell phone use, and it was utilized again in Study 2. Cronbach’s alpha was adequate (a = .75).

**Table 1.** Comparative view of frequency distributions of “yes” responses between AMPUH_sp_ (Study 1, student pop., *N* =301)and AMPUH_ap_ (Study 2, adult pop., *N* = 362)

Scored item	AMPUH_sp_		AMPUH_ap_	
	Count	%	Count	%
0	40	13.29	62	17.13
1	55	18.27	96	26.52
2	46	15.28	46	12.71
3	52	17.28	50	13.81
4	48	15.95	39	10.77
5	28	9.30	28	7.74
6	15	4.98	21	5.80
7	4	1.33	8	2.21
8	2	0.66	5	1.38
9	2	0.66	3	0.83
10	9	2.99	4	1.10
Total	301	100	362	100

*Note:* The AMPUHsp (student population) and the AMPUH_ap_ (adult population) items are based on DSM-IV (2000) criteria for”Pathologi-cal gamblingaddiction”.Scoringfive ormore items outoftenindicates problematic cell phone use. Sixty out of 301 (19.92%) participants responding to AMPUHsp scored five or more. Sixty-nine out of 362 (19.06%) participants responding to AMPUH_ap_ scored five or more.

### ACPAT results

The descriptive results for the ACPAT_sp_ (sp = student population) are as follows: *M* = 33.45, *SD* = 11.93, *SE* = 0.69, minimum = 20, maximum = 95. The overall results showed that 88.04% of the respondents scored within the low-to-moderate degree of concern category for problematic cell phone use (scoring between 20–49), 11.63% scored within the moderate-to-high category (scoring between 50–79), and 0.33% scored within the severe category (scoring between 80–100). Thus, approximately 12%, or 36 out of 301, of the entire sample size reflected a moderate-to-severe degree of concern relative to the addictive qualities of mobile phone use. Table 2 presents the frequency counts and percentages for the individual item indices and their sums (i.e., “Frequently” + “Often” + “Always” = Total). Notably, more than 20% of the respondents’ endorsements claim that they stay on their cell phones longer than intended, check their incoming messages before something else that they need to do, deprive themselves of sleep due to late-night phone use, and find themselves anticipating when they can use their cell phones. Less than 20% of the participants asserted that their grades or schoolwork suffer because of spending too much time on their phones, that their job performance and productivity suffer because of their cell phone use, and that others complain to them about the amount of time they spend on their mobile phones. With a Cronbach’s alpha of .93, the ACPAT possesses internal consistency, and its use was considered again in Study 2.

**Table 2.** Adapted cell phone addiction test (ACPAT) and comparative view of frequency distributions between student (ACPAT_sp_, *N* = 301) and nonstudent (ACPAT_ap_, *N* = 362) populations

	ACPAT_sp_	ACPAT_ap_
Item: How often...?	Count (%)	Count (%)
1. Staying on phone longer than intended.	124 (41.20)	109 (30.10)
2. Neglecting chores.	56 (18.60)	60 (16.60)
3. Preferring phone use to intimacy with partner.	23 (07.70)	49 (13.60)
4. Forming new relationships with callers.	54(18.00)	57 (15.80)
5. Others complaining about your phone use.	29 (09.60)	59 (16.30)
6. Grades/Schoolwork suffers.	32 (10.60)	35 (09.60)
7. Checking incoming messages first before other things.	191 (63.40)	143 (39.50)
8. Job performance/productivity suffers.	36(11.90)	54 (14.90)
9. Become defensive/secretive about phone use.	61 (20.30)	70 (19.40)
10. Using phone to block out other disturbing thoughts.	34(11.30)	57 (15.70)
11. Anticipating when you can use cell phone.	67 (22.30)	84 (23.20)
12. Fearing life without cell is boring/joyless.	51 (17.00)	72 (19.80)
13. Act annoyed when someone bothers you.	30 (09.90)	52 (14.30)
14. Losing sleep.	60 (20.00)	57 (15.80)
15. Preoccupied with being connected when cell is off.	43 (14.30)	52 (14.30)
16. Saying, “Just a few more minutes” when using cell.	49 (16.40)	68 (18.80)
17. Cutting down on cell phone use.	38 (12.70)	51 (14.10)
18. Inventing excuses to others why you’re on cell too long.	23 (07.60)	44 (12.20)
19. Choosing cell use over spending time with others.	24 (08.00)	47 (12.90)
20. Feeling depressed, moody or nervous when not using.	33 (10.90)	57 (15.80)

*Note:* ACPAT is a 20-item scale measuring degrees of concern relative to preoccupation (salience), excessive use, neglecting work/and social life, lack of control, and anticipation. Each item is semantically modified to fit cell phone use. Counts and percentages are summed scores of indices (i.e., “Frequently + Often + Always” = Total). Originally developed by Young (1998, 2004) for measuring Internet addiction and based on DSM-IV (2000) criteria for “pathological gambling”. ACPAT_sp_ (sp = student population), *a* = .93. ACPAT_ap_ (ap = adult population), *a* = .96.

## Study 1 Discussion

Study 1 achieved its purpose of measuring appetitive behavioral qualities relative to problematic mobile phone use. The AMPUH and ACPAT clearly showed that approximately 10 to 20% of the college students tested exhibited problematic cell phone use. More so, both the AMPUH and ACPAT provided statistical evidence for a number of criteria linked to problematic mobile phone use, thus substantiating findings similar to Young’s Internet addiction diagnostic questionnaires (Young, 1998). Most college students appear to show a preoccupation with their cell phones, satisfying the criterion for salience. They also use their cell phones to help them feel relaxed, escape problems, and lift their moods, fulfilling the criterion for escapism (or dysphoric relief) and mood modification. Explaining the factor of neglecting work, approximately 10% of the sample population of college students endorsed that their grades and schoolwork suffer because of the amount of time they spend on their phones. Additionally, a little over 10% of the sample perceive themselves as lacking some form of control when attempting to cut down the amount of time they spend on their phones and fail, and 50% of them find that they stay on their cell phones longer than they intended, achieving the criterion for lack of control. Many students (more than 50%) also prioritize the use of their cell phones before other pressing and important things they need to do (e.g., checking e-mail messages), satisfying the criterion for anticipation. Thus, an overall notable finding in Study 1 is the psychometric ability of the AMPUH and ACPAT in assessing problematic mobile phone use.

One limitation in Study 1 is the inability to generalize the findings gathered from the AMPUH and ACPAT’s to the general population. Because a convenience sampling method was used (i.e., the majority of the participants were students majoring in psychology), targeting the general population (i.e., the adult population) was an important consideration in Study 2. Thus, further testing of the AMPUH and ACPAT scales was utilized in Study 2 for their retest psychometric capabilities.

## Study 2 Method

### Study 2 participants

A total of 362 participants (194 men, 168 women, *M*_age_ = 32, age range: 18–74), comprised primarily of working adults, responded to a set of inventories via an online survey process. Out of the 379 respondents, 362 responses were retained for analysis. Seventeen respondents were excluded because of failing to complete all of the items on the questionnaires as required (response rate = 95.51%). A total of 8 inventories were used: the AMPUH (the identical questionnaire used in Study 1, see Appendix A), the ACPAT (the identical questionnaire used in Study 1, see Appendix B), the Mobile Phone Problem Use Scale (MPPUS; Bianchi & Phillips, 2005), Zung’s Self-Rating Depression Scale (ZSDS; Zung, 1965), Extraversion Scale, Emotional Stability Scale (ES), Rosenberg’s Self-Esteem Scale (RSES; Rosenberg, 1965), and the Impulse Control Scale (ICS). The extraversion, emotional stability and impulse control scales were taken from the International Personality Item Pool (IPIP) website, a public domain inventory bank. An evaluation of the linear relationships between depression, extra-version, emotional stability, self-esteem, impulse control, age, and the mean scores on the MPPUS and ACPAT were measured using Pearson’s correlation. A regression model analysis was also conducted using the mean scores for ACPAT and MPPUS as dependent variables, and using the mean scores for depression, extraversion, emotional stability, self-esteem, impulse control, and age as predictor variables. An inferential statistical test (i.e., *t*-test, chi-square) was also performed to test the AMPUH and ACPAT mean scores for equality across the two population samples.

*The Adapted Cell Phone Addiction Test (ACPAT_ap_).* A 20-item scale that was used in Study 1. The distribution was positively skewed (1.35) and close to normal (*M* = 32.63, *SD* = 14.57). With a Cronbach’s alpha of .96, the ACPAT_ap_ showed exceptionally high construct reliability and internal consistency.

*The Mobile Phone Problem Use Scale (MPPUS).* Developed by Bianchi and Phillips (2005), the 27-item MPPUS was designed to measure cell phone addiction. Each item is measured on a 10-point Likert scale. Summing all 27 items gives a total score, and the higher the score, the more concern is associated with problematic use. There are three categories of concern: scoring within the 27 to 76 range depicts a low-to-moderate degree of concern, scoring within the 77 to 126 range depicts a moderate-to-high degree of concern, and scoring within the 127 to 231 and above range depicts a high-to-severe degree of concern. Distribution was slightly positively skewed (0.74) and close to normal (*M* = 89.30, *SD* = 49.80). With a Cronbach’s alpha of .96, the MPPUS showed exceptionally high construct reliability and internal consistency.

*Zung’s Self-Rating Depression Scale (ZSDS).* Designed by Zung (1965), the ZSDS is comprised of 20 items and assesses the level of depression in individuals by quantifying the depressed status of the sufferer (*M* = 39.02, *SD* = 10.04). With a Cronbach’s alpha of .88, the ZSDS is a reliable scale in assessing levels of depression.

*Extraversion Scale.* The first component (Factor I) of the Big-Five Factor Markers for Surgency or Extraversion (Goldberg, 1992) consists of 10 items taken from the International Personality Item Pool (IPIP) website (*M* = 24.98, *SD* = 5.29). With an acceptable Cronbach’s alpha of .72, the Extraversion scale is a reliable inventory for assessing the extraverted trait.

*Emotional Stability Scale (ES).* The fourth component (Factor IV) of the Big-Five Factor Markers for emotional stability (1992) consists of 10 items (*M* = 34.81, *SD* = 9.26). With a Cronbach’s alpha of .93, the ES Scale is highly reliable for assessing the emotional stability trait.

*Rosenberg’s Self-Esteem Scale (RSES).* The Rosenberg Self-Esteem Scale (Rosenberg, 1965) consists of 10 items (*M* = 20.74, *SD* = 5.83). With a Cronbach’s alpha of .91, the RSES is highly reliable for assessing high and low levels of self-esteem. A participant scoring less than fifteen for their total score is considered as possessing low self-esteem.

*Impulse Control Scale (ICS).* Based on Cloninger’s temperament and character inventory (Cloninger, Przybeck, Svrakic & Wetzel, 1993), the ICS consists of 10 items (*M* = 34.07, *SD* = 7.34). With a Cronbach’s alpha of .87, the ICS is a reliable scale for assessing high and low levels of impulse control.

Two questions guided Study 2’s research: Are the AMPUH, ACPAT, and MPPUS scales reliable psychometric tools in addressing problematic cell phone use when wielded in the adult population, and can problematic cell phone use be predicted due to the susceptibility of an individual’s dispositional or behavioral traits?

### Ethics

The study procedures were carried out in accordance with the Declaration of Helsinki. The Institutional Review Board of San Francisco State University approved the study. All participants were provided an “informed consent” of the study.

## Study 2 Results

### AMPUH_sp_ and AMPUH_ap_ results

Presented in Table 1 are the comparative results between the AMPUH_sp_ (sp = student population) (*N* = 301) from Study 1 and the AMPUH_ap_ (ap = adult population) (*N* = 362) from Study 2. Substantiating that problematic cell phone use within the student population will show similar results to the adult population, the findings indicated that 60 out of 301 (19.92%) students and 69 out of 362 (19.06%) adults endorsed at least five or more criteria out of ten for problematic use.

An analysis testing for homogeneity determining whether the “yes” and “no” responses for each of AMPUH’s ten criteria taken from the two separate populations (i.e., student population and adult population) indicated retaining the null hypothesis (H_0_: The distribution of the bivariate categorical variable is the same across the populations). A chi-square test found the following criteria as nonsignificant: salience, *χ*^2^ (1, *N* = 663) = 0.07, *p* = .79; mood modification, *χ*^2^ (1, *N* = 663) = 0.00, *p* = .96; relapse, *χ*^2^ (1, *N* = 663) = 0.72, *p* = .40; withdrawal, *χ*^2^ (1, *N* = 663) = 2.78, *p* = .09; escapism/dysphoric relief, *χ*^2^ (1, *N* = 663) = 2.56, *p* = .11; tolerance, *χ*^2^ (1, *N* = 663) = 1.27, *p* = .26; cognitive dissension, *χ*^2^ (1, *N* = 663) = 2.36, *p* = .13; antisocial behavior, *χ*^2^ (1, *N* = 663) = 0.11, *p* = .74; conflict/loss, *χ*^2^ (1, *N* = 663) = 0.68, *p* = .41; and desperation, *χ*^2^ (1, *N* = 663) = 1.58, *p* = .21.

### ACPAT_sp_ and ACPAT_ap_ results

The ACPAT_ap_ (ap = adult population) showed internal consistency and high reliability as a scale addressing problematic cell phone usage. Problematic use within the student population showed similar results when those same scales were wielded in the general population. An unpaired *t*-test result indicated a nonsignificant outcome. Thus, the mean score of ACPAT_sp_ (*M* = 33.45, *SD* = 11.93, *N* = 301) was not significantly different from the mean score of ACPAT_ap_ (*M* = 32.63, *SD* = 14.57, *N* = 362), *t*(661) = 0.78, *p* = 0.43. The mean of ACPAT_sp_ minus the mean of ACPAT_ap_ equaled 0.82 at a 95% confidence interval [–1.24, 2.88]. Frequency counts and percentages for the individual item indices were summed (i.e., “Frequently” + “Often” + “Always” = Total). Table 2 depicts all 20 items and the notable frequency response outcomes for ACPAT_sp_ and ACPAT_ap_. The following are the comparative overall results between ACPAT_sp_ and ACPAT_ap_ frequency and classification categories for problematic cell phone usage concerns: For the student sample (*N* = 301), 265 (88.04%) students scored between 20–49 (i.e., showing a low-to-moderate degree of concern) and 36 (11.96%) students scored between 50–100 (i.e., showing a moderate-to-severe degree of concern). For the adult sample (*N* = 362), 306 (84.53%) showed a low-to-moderate degree of concern and 56 (15.47%) showed a moderate-to-severe degree of concern.

### ACPAT_ap_ and independent variables correlation results

The measures of the strengths of the linear relationships between the ACPAT_ap_ and six independent variables are presented in Table 3. A Pearson’s correlation coefficient indicated a significant linear relationship between depression and the ACPAT_ap_ responses: *r* = .43, *p* < 0.01. High scores on the ACPAT_ap_ showed a respectable negative linear association with emotional stability, *r* = –.31, *p* < 0.01; a moderately respectable negative linear association with self-esteem; *r* = –.27, *p* < 0.01, and a respectable negative linear association with impulse control, *r* = –.33, *p* < 0.01. Thus, the higher the respondents endorse themselves as being emotionally stable, having self-esteem, and having self-control, the fewer problems they have with their cell phone use. Extraversion, on the other hand, appeared to show a weak positive linear relationship with the ACPAT_ap_ score results, *r* = .14, *p* < 0.01. Age, as was expected, showed a respectable negative linear relationship with the ACPAT_ap_ scored results, *r* = –.35, *p* < 0.01. Thus, as age increases, fewer problems associated with cell phone usage were found.

### MPPUS results

With a Cronbach’s alpha of .96, the MPPUS showed internal consistency and high reliability as a scale addressing problematic cell phone usage (*M* = 89.30, *SD* = 46.80). The overall frequency score results showed that 50.28% of the respondents scored within the low-to-moderate degree of concern category for problematic use (scoring between 27–76), 24.31% scored within the moderate-to-high degree of concern category (scoring between 77–126), and 25.41% scored within the high degree of concern category (scoring between 127–231).

### MPPUS and independent variables correlation results

The measures of the strengths of the linear relationships between the MPPUS and six independent variables are presented in Table 3. A Pearson’s correlation coefficient indicated a significant linear relationship between depression and responses on the MPPUS, *r* = .45, *p* < 0.01. High scores on the MPPUS showed a respectable negative linear association with emotional stability, *r* = –.36, *p* < 0.01; a moderately respectable negative linear association with self-esteem, *r* = –.27, *p* < 0.01; and a respectable negative linear association with impulse control, *r* = –.39, *p* < 0.01. Thus, the higher the respondents endorsed themselves as being emotionally stable, having self-esteem, and having self-control, the fewer problems they had with their cell phone use. Extra-version, on the other hand, appeared to show a weak positive linear relationship with the MPPUS score results with *r* = .14, *p* < 0.01. Thus, results indicate that extraversion was not strongly associated with higher scores on the MPPUS. As expected, age showed a respectable negative linear relationship with the MPPUS score results, *r* = –.38, *p* < 0.01. Thus, cell phone use problems decrease as age increases.

### Analysis and results for predicting problematic cell phone use

Addressing the aim of this study that individuals possessing certain behavioral or dispositional traits are more susceptible to problematic mobile phone use, a linear regression model was used to predict problematic use as a function of age, depression, extraversion, emotional stability, impulse-control and self-esteem. The ACPAT_ap_ and MPPUS overall mean scores were considered as dependent variables, and age, depression, extraversion, emotional stability, impulse control and self-esteem mean scores were considered as independent variables (i.e., predictors) in the regression model analysis. Prior to forming the model that fit best and correcting for any unequal variances, all outliers were trimmed using transformations (i.e., a model summary was developed using the SPSSversion 20 automatic linear modeling engine). A forward stepwise model selection method was employed with a selected confidence level (CL) at 95% (*p* < 0.05).

**Table 3.** Correlations (parametric) for mean scores on the ACPAT_ap_, MPPUS, ZSDS, extraversion, emotional stability, self-esteem, age and impulse control inventories (*N* = 362)

Measure	1	2	3	4	5	6	7	8
1. ACPAT_ap_	(.96)							
2. ZSDS	.43	(.88)						
3. Extrav.	.14	–.27	(.72)					
4. ES	–.31	–.70	.25	(.93)				
5. RSES	–.27	–.68	.32	.59	(.91)			
6. ICS	–.33	–.66	.21	.63	.63	(.87)		
7. Age	–.35	–.28	<.01	.21	.17	.26		
8. MPPUS	(.96)	.45	.14	–.36	–.27	–.39	–.38	
Mean	32.63	39.02	24.98	34.81	20.74	34.07	32.63	89.30
*SD*	14.57	10.04	05.29	09.26	05.83	07.34	11.66	46.77

*Note:* Items in parenthesis are Cronbach’s alphas. ZSDS = Zung’s Self-Rating Depression Scale. ES = Emotional Stability Scale. RSES = Rosenberg’s Self-Esteem Scale. ICS = Impulse Control scale. The Adapted Cell Phone Addiction Test [ACPAT_ap_ (ap = adult population)] measures the degree (i.e., how often) of problematic cell phone usage. High scores on the ACPAT_ap_ indicate problem cell phone use. The Mobile Phone Problem Use Scale (MPPUS) measures the degree of problematic mobile phone use. High scores on the MPPUS indicate problematic cell phone use. *p* <0.01.

*ACPAT_ap_ regression analysis.* A multiple regression was conducted with the following predictor variables: age, ZSDS (i.e., depression), extraversion scale, emotional stability scale, ICS (i.e., impulse control), and RSES (i.e., self-esteem), with ACPAT_ap_ as the outcome variable. The model produced an R square of .306, which was statistically significant, *F*(5,356) = 26.07, *p* < 0.05. Age, depression, extraversion, emotional stability, impulse control, and self-esteem can account for 30.6% of the variance in ACPAT_ap_. According to Myers (1990), the variance inflation factor (VIF) that shows a value of less than ten is an acceptable number that checks for multicollinearity in the regression model. All predictor variables had VIF values of less than five, assessing an acceptable threshold for each predictor in the model. Depression, *β* = 0.37, [0.33, 0.75], *t* = –5.08, *p* < 0.05 and extraversion scales, *β* = 0.26, [0.46, 0.97], *t* = –5.52, *p* < 0.05 were positively related to ACPAT_ap_. The emotional stability scale, *β* = –0.05, [–0.25, 0.16], *t* = –0.47, *p* > .05, the ICS scale, *β* = –0.04, [–0.37, 0.13], *t* = –0.93, *p* > .05, age, *β* = –0.23, [–0.40, –0.17], *t* = –4.87, *p* < 0.05, and self-esteem, *β* = –0.00, [–0.33, 0.31], *t* = –0.05, *p* > .05 were negatively related to ACPAT_ap_. Thus, the most suitable predictors in the regression model were depression, extraversion, and age, with depression being the strongest predictor (i.e., of importance) followed by extra-version and age. Goodness-of-fit was determined using semi-partial correlations in determining the percentage of variability of each independent variable in the regression model: depression accounted for 5%, extraversion accounted for 6%, and age accounted for 5% of the variability. Emotional stability, impulse control, and self-esteem were nonsignificant, *p* > .05; therefore, their effects did not enter into the ACPAT_ap_ regression model.

*MPPUS regression analysis.* Again, predicting problematic mobile phone use, a multiple regression was conducted with the following predictor variables: age, depression, extraversion scale, emotional stability scale, impulse control, and self-esteem, with MPPUS as the outcome variable. The model produced an R square of .351, which was statistically significant, *F*(5,356) = 31.90, *p* < .05. Age, depression, extraversion, emotional stability, impulse control, and self-esteem can account for 35.10% of the variance in MPPUS. Again, according to Myers (1990), the variance inflation factor (VIF) that shows a value of less than ten is an acceptable number that checks for multicollinearity in the regression model. All predictor variables had VIF values of less than five, assessing an acceptable threshold for each predictor in the model. Age, *β* = –0.24, [–1.31, –0.60], *t* = –5.32, *p* < .05, emotional stability, *β* = –0.07, [–1.01, 0.27], *t* = –1.15, *p* > .05, and impulse control, *β* = –0.13, [–1.61, –0.04], *t* = –2.05, *p* = 0.041 were negatively related to MPPUS. Depression, *β* = 0.35, [0.99, 2.29], *t* = 4.98, *p* < .05, the extraversion scale, *β* = 0.27, [1.55, 3.14], *t* = 5.83, *p* < .05 and self-esteem, *β* = 0.05, [–0.63, 1.36], *t* = 0.72, *p* > .05 were positively related to MPPUS. Thus, the most suitable predictors in the regression model were depression, extra-version, age, and impulse-control, with depression being the strongest predictor (i.e., of importance) followed by extraversion, age, and impulse control. Goodness-of-fit was determined using semi-partial correlations in determining the percentage of variability of each independent variable in the regression model. Depression accounted for 5%, extra-version accounted for 6%, age accounted for 5%, and impulse control accounted for 1% of the variability in the regression model. Emotional stability and self-esteem were nonsignificant, *p* > .05; therefore, their effects did not enter into the MPPUS regression model.

### Age and problematic use

Addressing age, older mobile phone users reported fewer problems relative to their scores on the AMPUH_ap_, ACPAT_ap,_ and MPPUS.

*AMPUH_ap_ and age results.* Categorized into four age groups ranging from youngest to oldest (i.e., 18–29, 30–39, 40–49, and 50 >), the responses on each of AMPUH_ap_’s 10 items clearly showed that as the age category increases, the “yes” response frequency counts (observed) endorsing problematic use decreases on at least eight of the ten criteria (see Table 4). Chi-square tests for each of the factors, testing for equality across the four age groups, showed salience, *χ*^2^ (3, *n* = 140) = 32.71, *p* < .05; mood modification, *χ*^2^ (3, *n* = 164) = 29.55, *p* < .05; withdrawal, *χ*^2^ (*n* = 70) = 27.55, *p* < .05; escapism, *χ*^2^ (*n* = 144) = 23.67, *p* < .05; cognitive distortion, *χ*^2^ (*n* = 38) = 19.35, *p* < .05; antisocial behavior, *χ*^2^ (*n* = 19) = 11.76, *p* < .05; conflict/loss, *χ*^2^ (*n* = 21) = 10.70, *p* < .05; and desperation, *χ*^2^ (*n* = 32) = 17.85, *p* < .05, were not equal across the four age groups. Thus, as younger individuals reported more problems associated with their mobile phone use, older individuals endorsed fewer.

*ACPAT_ap_ and age results.* Categorizing four age groups ranging from youngest to oldest (i.e., 18–29, 30–39, 40–49, and 50 >), a one-way analysis of variance (ANOVA) was performed testing the hypothesis that the average mean scores of the ACPAT_ap_ across the four age groups would be equal. The results indicated that the average scores were found to be different across the four age groups, *F*(3, 358) = 22.16, *p* = .000. The Tukey multiple comparisons performed at the 0.05 significance level found that the mean ACPAT_ap_ score in the 18–29 age group (*M* = 37.97, *SD* = 15.94, *N* = 187) was significantly higher than that for the 50 and above age group (*M* = 23.40, *SD* = 3.78, *N* = 46), the 30–39 age group (*M* = 27.40, *SD* = 10.22, *N* = 90), and the 40–49 age group (*M* = 30.00, *SD* = 13.98, *N* = 39).

Following suit, a one-way ANOVA was also performed on the MPPUS scores, testing for equality across the four age groups. The results indicated that the average scores were found to be significantly different across the four age groups, *F*(3, 358) = 24.55, *p* = .000. The Tukey multiple comparisons performed at the 0.05 significance level found that the mean MPPUS score in the 18–29 age group (*M* = 106.83, *SD* = 47.60, *N* = 187) was significantly higher than that for the 50 and above age group (*M* = 55.49, *SD* = 19.85, *N* = 46), the 30–39 age group (*M* = 73.56, *SD* = 36.98, *N* = 90), and the 40–49 age group (*M* = 81.49, *SD* = 49.77, *N* = 39). Thus, as the ages increased, fewer cell phone problems were endorsed on the ACPAT_ap_ and MPPUS scales.

**Table 4.** Chi-square test results for age categories and AMPUH_ap_ criteria (*N* = 362)

	Frequency counts of “yes” responses in age category					
Criterion	18–29	30–39	40–49	50 >	*χ*^2^	*^p^*
1. Salience	98	26	8	8	32.71	< .05
2. MM	110	31	10	13	29.55	< .05
3. Relapse	49	18	9	5	5.39	ns
4. Withdrawal	55	8	6	1	27.55	< .05
5. Escapism/DR	96	29	10	9	23.67	< .05
6. Tolerance	113	53	22	26	0.38	ns
7. CD	32	3	3	0	19.35	< .05
8. ASB	17	1	1	0	11.76	< .05
9. Conflict/Loss	18	2	1	0	10.70	< .05
10. Desperation	26	1	5	0	17.85	< .05

*Note:* Degrees of freedom = 3. MM = mood modification; DR = dysphoric relief; CD = cognitive distortion; ASB = antisocial behavior. Confidence interval set at 95%, ns denotes nonsignificantp-value. As the age category increases, participants responding to each criterion with a “yes” response become fewer. Thus, older aged individuals endorse fewer problems associated with their cell phone use.

## Discussion

**Figure 1.**
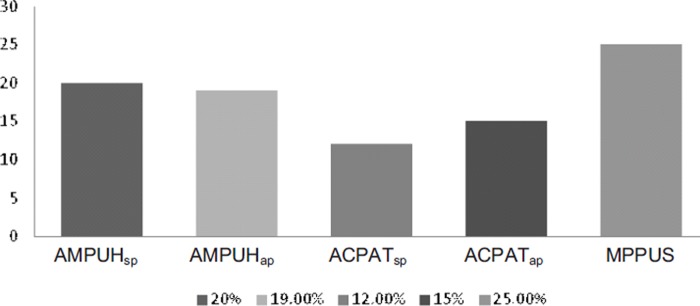
Study 1 (student pop., *N* = 301) and Study 2 (adult pop., *N* = 362) summary results for problematic mobile phone usage. The AMPUH_sp_ and ACPAT_sp_ are inventories used at SFSU (student population). The AMPUH_ap_, ACPAT_ap_ and MPPUS were inventories used in the general population (adult population). The AMPUH frequency scores are based on the DSM-IV (2000) criteria for behavioral addiction; whereby, endorsing five or more items out of ten express problematic use. ACPAT results are based on a moderate-to-severe category for dependency: The higher the score on the ACPAT_sp,_ and ACPAT_ap_, the higher the degree of dependency. The MPPUS score (approximately 25% of cell phone users) denotes a high concern relative to problematic cell phone usage

Study 1 and Study 2 achieved the objectives of measuring problematic mobile phone use. The utilization of the AMPUH, ACPAT and MPPUS scales clearly showed that college students, as well as nonstudents, exhibited problematic cell phone use and their behavior satisfied a number of the addiction criteria. Figure 1 illustrates the findings for the prevalence of problematic use and the measured degree of its concern as evidenced by the two population samples tested.

Consistent with previous research (Bianchi & Phillips, 2005; Gentile, 2009; Griffiths, 1995; Young, 1998, 2004), discrete components such as preoccupation, escapism, dysphoric relief, mood modification, tolerance, anticipation, lack of control, neglecting work and social life, and excessive use were psychometrically evident in all three of the inventories addressing problematic cell phone use. Additionally, the self-reported responses coincide with this study’s proposed theoretical model addressing problematic cell phone use: over 40% of the respondents use their cell phones to help them feel relaxed, as well as to escape problems and lift their moods. Thus, a tentative conclusion can be drawn that users moderate their moods by engaging in cell phone activity, showing a coping mechanism at work; however, as previously mentioned, this model is not an absolute – using only coping/learning effects in addiction is cautioned, for there are other principles that explain addiction that may have added culpability. Thus, advancing explanatory models that minimize the difficulty in explaining all phenomena associated with problematic cell phone use is of immense scientific importance for addiction researchers, as well as for physicians.

Since the AMPUH and ACPAT items were semantically modified to fit cell phone use, testing the reliability of both inventories in the nonstudent population was of statistical interest. The frequency counts and percentages across both AMPUH samples demonstrated consistent endorsements. Substantiating previous research studies utilizing inventories with scales taken from the DSM’s criteria for impulse control disorder (Grant et al., 2010; Young, 1998), the AMPUH_sp_ and AMPUH_ap_ scales clearly evidenced that approximately 20% of both the student and adult samples endorsed five or more criteria out of ten, underpinning the DSM’s (2000) specifications for behavioral addiction. Testing the AMPUH_sp_ and AMPUH_ap_ across both population samples for homogeneity and examining the value differences between the “yes” and “no” responses, the sampling results indicated a considerably small difference between the observed and expected counts. Thus, chi-square tests evinced retaining the null hypothesis (i.e., the distribution of the bivariate categorical variable is the same across the populations).

ACPAT’s psychometric capabilities performed as expected across both population samples. According to Young (1998) and the development of her Internet Addiction Test (IAT), the ACPAT scale is highly reliable in addressing Internet addiction. Thus, this study poses a question for future research: How well will the ACPAT, with its modified items addressing problematic cell phone use, perform psychometrically? Based on the results of the present study, it appears that the ACPAT has *potential* as a reliable scale addressing problematic use relative to cell phone use. Showing substantiating psychometric capabilities, the AMPUH and ACPAT endorsed the objective that utilizing inventories across the college student and nonstudent populations, problematic cell phone use can be measured.

In estimating the strength of the relationships between the ACPAT_ap_, MPPUS scores and the six independent variables (i.e., age, self-esteem, extraversion, emotional stability, depression, and impulse control), all the variables showed significant linear relationships with both dependent variables (ACPAT_ap_ and MPPUS scores).

Estimating the predictive utility of both the ACPAT_ap_ and the MPPUS, the results partially support Bianchi and Phillips’ (2005) findings. Lessened emotional stability (i.e., neuroticism) and low self-esteem, it appears, did not predict higher scores on the ACPAT_ap_ and MPPUS. One explanation may be linked to the type of use contributed by mobile phone users. Past research (Amiel & Sargent, 2004) indicated that neurotics prefer to do less interactive socializing on their mobile phones (e.g., less e-mailing and texting), which may be one reason why neuroticism does not predict higher scores on the ACPAT_ap_ and MPPUS regression models. Surprisingly unexpected, individuals with low self-esteem did not have an effect on predicting problematic use on both the ACPAT_ap_ and MPPUS, while impulse control had an effect on only the MPPUS scale. One speculative reason for low self-esteem’s shortfall in predicting higher scores on both inventories may be linked to the types of cell phone activities that low self-esteem individuals perform. It is quite possible that only highly interactive activities result in problematic use, such as gaming and chat rooms. Based on the predictive results shown in this study, low self-esteem users may not consider social network sites (SNS; e.g., Facebook) as an appealing activity, as Forest and Wood (2012) had claimed. Preferring e-mailing, “surfing,” and less interactive apps may be more appealing for low self-esteem individuals. Another theoretical explanation may be related to the measurement of self-esteem itself and its predictive utility, that is, the effects of self-esteem often coincide with the effects of other correlated variables, such as depression, and the apparent effects of self-esteem disappear when controlling for other variables (Baumeister et al., 2003). Previous research has shown that individuals with low self-esteem express the need for reassurance from others (Baumeister et al., 2003), but this does not necessarily mean they actively seek it out via their mobile phones (i.e., through active participation on SNS). Perhaps the variability in problematic cell phone use endorsements by people with low self-esteem is based on their need for reassurance and the contextual motivation to seek it out. Facebook relationships, for example, may not be such an appealing venue for seeking reassurance because of unfavorable responses from others (i.e., low self-esteem individuals tend to accentuate the negative); thereby, individuals with low self-esteem reduce their amount of logging on to social network sites.

As expected, depression, extraversion, and age predicted higher scores on both the ACPAT_ap_ and MPPUS inventories. Noting also, even though extraversion appeared to show a weak positive relationship in the correlation analysis, it did have a significant effect in predicting higher scores on the ACPAT_ap_ and MPPUS regression models. Overall, problematic cell phone use can be predicted, and according to the current regression models, is a function of age, depression, extraversion, and impulse control. Confirming earlier studies (Young & Rogers, 1998; Lam & Peng, 2010; Morrison & Gore, 2010) that depressed individuals tend to frequent the Internet much more than those without symptoms, depression, it appears, has the strongest association with problematic mobile phone use; followed by extraversion, age, and impulse control.

### Limitations

This study did not address whether or not respondents had smartphones or access to the Internet. Future studies should include these demographic technologies. Additionally, this study does not address the diverse types of mobile phone use, such as whether the user endorses essential or non-essential use, or both. Underscoring the varied subjective meanings, dependency for some users was based on work-related reasons for the mobile phone, making dependency an essential use. Others used their mobile phones for staying in constant communication with either family or friends – this can also be considered essential use. Still others used their mobile phones for entertainment (e.g., taking and sending pictures, gaming, and watching movies) – this too can be considered essential, while others may find it wasting time. Therefore, the above-mentioned discrepancies make it difficult to categorize what essential and nonessential mobile phone use connotes, and further investigation into these two factors was not explored in this study. Future studies may opt for developing sensitivity scales that effectively address essential and nonessential use; for the type of use may reflect the motivational behaviors of users, as well as addressing the ambiguousness of dependency. Another study limitation that may need addressing from future researchers is exploring the dimensionality (latent variables) of the AMPUH and ACPAT scales. This study did not conduct a factor analysis on these two inventories and researchers should opt to investigate their dimensionality. By increasing the sample size and using a Likert scale for the AMPUH scale, rather than a dichotomized one, a factor analysis would provide psycho-metric support for the AMPUH as well as the ACPAT scales as distinct constructs.

## Conclusion and Future Directions

Among the scientific literature, many traditional theoretical models attempting to explain virtual addictions are limited in scope – at least presently, it appears that ICT excessive behaviors are multifaceted (Suratt, 2006). Many traditional theoretical models explaining TA are varied, and most, if not all, address behavioral addictions with etiologies paralleling chemical addiction. In other words, some clinicians’ diagnostic criteria for assessing behavioral addictions are presently based on the traditional medical model or “germ theory” of diseases (Suratt, 2006). Based on past studies, there is no denying that some Internet users develop into “problematic users” who become addicted to social networking, gaming and buying (Roberts & Pirog, 2013; Shapira et al., 2003; Young, 2004). Using a conservative figure, on average, approximately 6% of Americans, who frequent the internet, suffer from Internet addiction (Greenfield, 1999). With the relatively new condition of mobility and Internet access convenience (i.e., individuals no longer have to wait to go home and sit at computers to access the Web), is it safe to assume – based on this study’s results – that the percentage of cell phone addicts has increased? Additionally, should the user exhibiting problematic cell phone use be considered a distinct disorder; or is it consequential, due to the presence of co-occurring primary disorders such as mood, depressive, or anxiety disorders (Shapira et al., 2003), or mediated by a high materialism trait (Roberts & Pirog, 2013)? Even more so, is the addition of Internet accessibility in cell phone applications responsible for more problematic cell phone use? Ongoing empirical research that addresses these issues, over time, may provide the psychological community – especially the framers of the DSM Task Force – with predictive capabilities in endorsing a positive response to what Griffiths (2000) initially speculated: Does information and communication technology addiction [actually] exist? Based on this study’s results, problematic mobile phone use does occur and ought to be taken seriously by the psychological community, as well by researchers investigating behavioral addictions. Presently, there is limited data providing conclusive evidence for a comprehensible categorization of cell phone addiction, as well as a unified explanatory model specific to problematic mobile phone use. Studies such as this one may contribute substantial findings, adding scientific significance, and offering a valuable submission for the ongoing progress of creating intervention frameworks relative to “virtual addictions”. Owing to the condition of Internet access mobility, the findings from this present study encourage the continued empirical research of the interactions between cell phone use and diagnostic criteria.
